# Impact of social support on oral health among immigrants and ethnic minorities: A systematic review

**DOI:** 10.1371/journal.pone.0218678

**Published:** 2019-06-20

**Authors:** Rana Dahlan, Ebtehal Ghazal, Humam Saltaji, Bukola Salami, Maryam Amin

**Affiliations:** 1 School of Dentistry, University of Alberta, Edmonton, Alberta, Canada; 2 School of Dentistry, King Abdulaziz University, Jeddah, Saudi Arabia; 3 Faculty of Nursing, University of Alberta, Edmonton, Alberta, Canada; Erasmus Medical Center, NETHERLANDS

## Abstract

**Objective:**

Adaptation to social life changes after migration may be beneficial or detrimental to migrants’ oral health outcomes and related behaviors. This systematic review aims to synthesize the scientific literature on the impact of social support on immigrants’ and ethnic minorities’ oral health status and/or behaviors.

**Methods:**

A comprehensive electronic search, up to November 2018, was conducted using five electronic databases. We included cross-sectional and longitudinal quantitative studies that examine associations between social support and oral health outcomes among immigrants and ethnic minorities. Study selection, data extraction, and risk of bias assessment were completed in duplicate and the Newcastle-Ottawa checklist was used to appraise the methodological quality of the quantitative studies.

**Results:**

A total of 26 studies met the eligibility criteria. Included studies examined multiple oral health outcomes such as dental care utilization, oral health behaviors, oral health problems, self-rated oral health, oral health knowledge, and oral health-related quality of life among immigrants and ethnic minorities. The social support level is assessed either by social support indicators or by adapting certain scales. Overall, social support was found to be positively associated with dental care utilization, number of carious teeth, periodontal disease, oral health behaviors, oral health knowledge, oral health-related quality of life, and self-rated oral health.

**Conclusion:**

Although immigrants and ethnic minorities encounter several challenges after migration to a new country that could affect their oral health, social support from their surrounding environment in the form of structural or functional support plays an important role in improving their oral health outcomes.

## Introduction

Post-migration challenges, including language and cultural barriers, housing, employment, poverty, and lack of medical and dental insurance, may affect immigrants’ and ethnic minorities’ general and oral health [1, 2, 3]. Additionally, social life changes such as a decreased number of close family members and friends may lead to isolation and loneliness among immigrants and ethnic minorities [1, 2], which can consequently affect immigrants’ health and social life negatively due to the adoption of detrimental habits as coping methods [2, 3]. Therefore, the level of social support within the host country plays a crucial role in general and oral health outcomes among ethnic minorities.

Migration is a social determinant of health including oral health [4]. A higher level of dental diseases and underutilization of dental care have been reported among immigrants as compared to their native-born counterparts [5, 6], specially among those who had lived in the host country for less than 10 years [7, 8]. In addition, while many native-born children see dentists for preventive purposes, immigrants receive less preventative services and more treatments [9]. Therefore, disparities in oral health among immigrants is a serious public health matter that should not be neglected.

Social support has been defined variously as support "provided by other people [that] arises within the context of interpersonal relationships" [10, 11] and support which is "accessible to an individual through social ties to other individuals, groups, and the larger community" [11, 12]. Moreover, social support was defined as the social resources that people perceive to be available to them or that are actually offered either by formal support groups or informal relationships. [13] Therefore, social support can be perceived or received. The received social support is the actual support received by individuals. [14] While the perceived social support is “The individual's beliefs about the availability of varied types of support from network associates”.[14]

House and Cobb [15, 16] divided the notion of social support into four distinct types–emotional, instrumental, informational and appraisal; this approach has been widely adopted by many researchers as a method for measuring social support levels [11, 15, 16]. Social support can also be defined by applying the two broad categories of structural and functional measures [17]. The structural measures of social support include elements such as strength of social connections, frequency of social contact, and size and characteristics of a social network. [17–19] The functional measures of social support are further divided into four components: 1) emotional/moral support, which involves expressions of empathy, love, trust and care from family members and friends; 2) instrumental support, which provides services such as transportation or financial assistance; 3) informational support, which provides information and suggestions that help with problem solving; and 4) appraisal support, which provides self-evaluation, affirmation, and feedback [17–19].

In attempting to find associations between social support and general health, a number of studies discovered that social support was positively associated with mental health, heart attack survival, cancer reoccurrence, psychological problems as depression, and subjective well-being in breast cancer patients [3, 20–23]. Similar correlations have been reported in oral health. Dental care utilization among Latino immigrants’ children increased remarkably when social support was provided by family members or friends in different forms, such as booking an appointment (instrumental aid and influence), getting to the dentist (material aid), and accompanying them to the appointment (emotional aid) [18]. However, the provision of dental care information (instrumental aid) to Latino mothers by dentists or health care providers was not significantly associated with frequency of dental visits in children [18]. Social support provided by family members and friends was also a significant factor in receiving dental care among elderly Chinese immigrants living in the United States [24].

To the best of our knowledge, there is no systematic review that synthesize the existing evidence on associations between social support and oral health. Therefore, the purpose of the present report is to systematically review the available scientific literature on the impact of social support on immigrant and ethnic minorities’ oral health outcomes.

## Methods

### Protocol and registration

The systematic review protocol was registered at PROSPERO (registration number CRD42018095199; http://www.crd.york.ac.uk/prospero/), and the review has been conducted and reported in accordance with the Cochrane Handbook [25] and the Preferred Reporting Items for Systematic Reviews and Meta-Analyses (PRISMA) statements for reporting systematic reviews of health sciences [26].

### Eligibility criteria

We included cross-sectional and longitudinal quantitative studies that met the following predefined inclusion criteria: 1) examined the associations between social support and at least one oral health problem (such as dental caries, periodontal disease, self-reported oral pain, denture problems, sore or bleeding gums, and dry mouth) or oral health behaviors (such as dental care utilization, brushing, flossing, or diet); 2) included a clearly-defined measure of social support and a well-described assessment tool for oral health status or behaviors; and 3) were conducted with at least one immigrant or ethnic group; a group of people of a particular race or nationality, who have different ethnicity or culture from that of the majority in a certain country. Excluded from this study were literature reviews, conference abstracts, and editorials. No restrictions were applied on age, sex, or socioeconomic status.

### Data sources and search strategy

A comprehensive electronic search was conducted up to November 2018 using the following electronic bibliographic databases: Ovid MEDLINE (1946 to November, 2018), Ovid PsychInfo (1806 to November 2018, week 1), Sociological Abstracts (1988 to 2018), Embase (1974 to November, 2018), and Cinahl (2013–2018) (Table 1). The search strategy was developed with the assistance of a specialized health sciences librarian at the University of Alberta, Edmonton, Canada. The search terms included the following: “oral health”, “dental health”, “dentist”,” periodontal disease”, “tooth diseases”, “dental caries”, “immigrant”, “ethnic groups”, “immigration”, “social support”, “social network”, “social relationships”, “social integration”, “social connectedness”, and “social tie”. Table 1 provides details on the specific search terms and combinations used in each individual database. Manual screening was completed by searching through bibliographies and reference lists of the included papers to determine potential papers that were not found in the electronic search. Finally, a grey literature search was conducted by using the Google Scholar and Google search engines. All references resulting from the searches were exported to EndNote X8.2, and within this program, duplicates were electronically removed.

**Table 1:** Search strategy and results from different electronic databases.

Database	Keywords	Results
Ovid MEDLINE 1946—Nov 2018	dental health.mp. OR oral health.mp. OR exp Oral Health/ OR dentist*.mp. OR exp Dentists/ OR exp Periodontal Diseases/ OR periodontal disease*.mp. OR tooth disease*.mp. OR dental caries.mp. or exp Dental Caries/ AND emigrant*.mp. OR immigrant*.mp. OR exp "EMIGRANTS AND IMMIGRANTS"/ OR exp Ethnic Groups/ OR ethnic group*.mp. OR emigration.mp. OR exp "Emigration and Immigration"/ OR immigration.mp. OR migrant*.mp. AND exp Social Support/ OR Social support*.mp.OR exp Social Networking/ OR Social Networking*.mp. OR social network*.mp. OR Social relationships.mp. OR social tie*.mp. OR social integration*.mp. OR social connectedness.mp. OR social support network*.mp.	57
PsychInfo 1806—Nov 2018	dental health.mp. OR oral health.mp. OR exp Oral Health/ OR dentist*.mp. or exp Dentists/ OR exp Periodontal Diseases/ OR periodontal disease*.mp. OR tooth disease*.mp. OR dental caries.mp. OR exp Dental Caries/ AND emigrant*.mp.OR immigrant*.mp. OR exp "EMIGRANTS AND IMMIGRANTS"/ OR exp Ethnic Groups/ OR ethnic group*.mp. OR emigration.mp. OR exp "Emigration and Immigration"/ OR immigration.mp. OR transient*.mp. OR migrant*.mp. AND exp Social Support/ OR Social support*.mp. OR exp Social Networking/ OR Social Networking*.mp. OR social network*.mp. OR Social relationships.mp. OR social tie*.mp. OR exp Interpersonal Relations/ OR social engagement*.mp. OR social integration*.mp. OR social connectedness.mp. OR social support network*.mp.	20
Sociological Abstracts 1988—Nov 2018	(noft(dental health) OR noft(oral health) OR MAINSUBJECT.EXACT.EXPLODE("Dentists") OR noft(dentist*) OR noft(periodontal disease*) OR noft(tooth disease*) OR noft(dental caries) OR noft(caries) OR noft(tooth decay)) AND (noft(emigrant*) OR noft(immigrant*) OR noft(emigration) OR noft(immigration) OR noft(transient*) OR noft(migrant*) OR noft(ethnic group*) OR MAINSUBJECT.EXACT.EXPLODE("Emigration") OR MAINSUBJECT.EXACT.EXPLODE("Migration") OR MAINSUBJECT.EXACT.EXPLODE("Immigrants") OR MAINSUBJECT.EXACT.EXPLODE("Emigration") OR MAINSUBJECT.EXACT.EXPLODE("Immigration") OR MAINSUBJECT.EXACT.EXPLODE("Migrants") OR MAINSUBJECT.EXACT.EXPLODE("Ethnic Groups")) AND (noft(Social Support*) OR noft(Social Networking*) OR noft(social network*) OR noft(Social relationship*) OR noft(social tie*) OR noft(social engagement*) OR noft(social integration*) OR noft(social connectedness) OR noft(social support network*) MAINSUBJECT.EXACT.EXPLODE("Social Networks") OR MAINSUBJECT.EXACT.EXPLODE("Social Relations") OR MAINSUBJECT.EXACT.EXPLODE("Social Integration") OR MAINSUBJECT.EXACT.EXPLODE("Social Support"))	29
Embase 1974—Nov 2018	dental health.mp. OR oral health.mp. OR exp Oral Health/ OR dentist*.mp. OR exp Dentists/ OR exp Periodontal Diseases/ OR periodontal disease*.mp. OR tooth disease*.mp. OR dental caries.mp. OR exp Dental Caries/ AND emigrant*.mp. OR immigrant*.mp. OR exp "EMIGRANTS AND IMMIGRANTS"/ OR exp Ethnic Groups/ OR ethnic group*.mp. OR emigration.mp. OR exp "Emigration and Immigration"/ OR immigration.mp. OR transient*.mp. OR migrant*.mp. AND exp Social Support/ OR Social support*.mp. OR Social Networking/ or Social Networking*.mp. OR social network*.mp. OR Social relationships.mp. OR social tie*.mp. OR exp Interpersonal Relations/ OR social engagement*.mp.OR social integration*.mp. OR social connectedness.mp. OR social support network*.mp.	3424
CINAHL 2013—Nov 2018	(MH "Dental Caries") OR (MH "Oral Health (Omaha)") OR (MH "Oral Health (Iowa NOC)") OR (MH "Oral Health") OR "dental health OR oral health OR dentist* OR periodontal disease* OR tooth disease* OR dental caries OR caries OR tooth decay" AND (MH "Emigration and Immigration") OR (MH "Immigrants+") OR (MH "Ethnic Groups+") OR "emigrant* OR immigrant* OR emigration OR immigration OR transient* OR migrant* OR ethnic group*" OR (MH "Minority Groups") AND (MH "Social Support (Iowa NOC)") OR (MH "Social Networks") OR (MH "Social Networking") OR (MH "Social Involvement (Iowa NOC)") OR (MH "Social Interaction Skills (Iowa NOC)") OR (MH "Social Interaction (Iowa NOC)+") OR "Social Support* OR Social Networking* OR social network* OR Social relationship* OR social tie* OR Interpersonal Relation* OR social engagement* OR social integration* OR social connectedness OR social support network*"	5
Total databases searches		3535
Duplicates		180
Final		3355

### Study selection

Two reviewers (RD and EG) independently screened the list of titles and abstracts to identify potentially relevant papers based on the inclusion criteria. If the abstracts were judged to contain insufficient information, the full articles were reviewed to decide whether or not they should be included based on the selection criteria. When a discrepancy in the selection decision occurred, the two reviewers discussed how to deal with the discrepancy until a consensus was reached.

### Data extraction and data items

Two reviewers (RD and EG) independently extracted data from the selected papers on the following items: host country, participants’ origins and ages, sampling, sample size, type of study, social support measure, association with oral health outcomes, and results. Inconsistencies were discussed and resolved between the two authors. Missing or unclear information was sought from the authors of the selected papers.

### Risk of bias in individual studies

Two reviewers (RD and EG) independently assessed the methodological quality of the selected studies by using the Newcastle-Ottawa Scale [25] for cohort and cross-sectional studies, by scoring the three main categories of group selection (four items), comparability (one item), and outcome (two items) [27]. A study was awarded a maximum of five stars for selection, a maximum of two stars for group comparability, and a maximum of three stars for outcome categories. The highest methodological quality was indicated by the maximum score (10 points). Studies scoring less than 3 were considered low quality, while those scoring between 3 and 8 were considered medium quality and those above 8 were considered high quality. Although the NOS is an easy-to-apply and adaptable tool, it has some limitations in that there is no manual tool to use as a guide and it is not validated for cross-sectional studies [28, 29].

### Synthesis of results

Due to the heterogeneity of the included studies, the findings were evaluated in a descriptive manner. It was not possible to conduct a meta-analysis.

## Results

### Study selection

**Fig 1:**
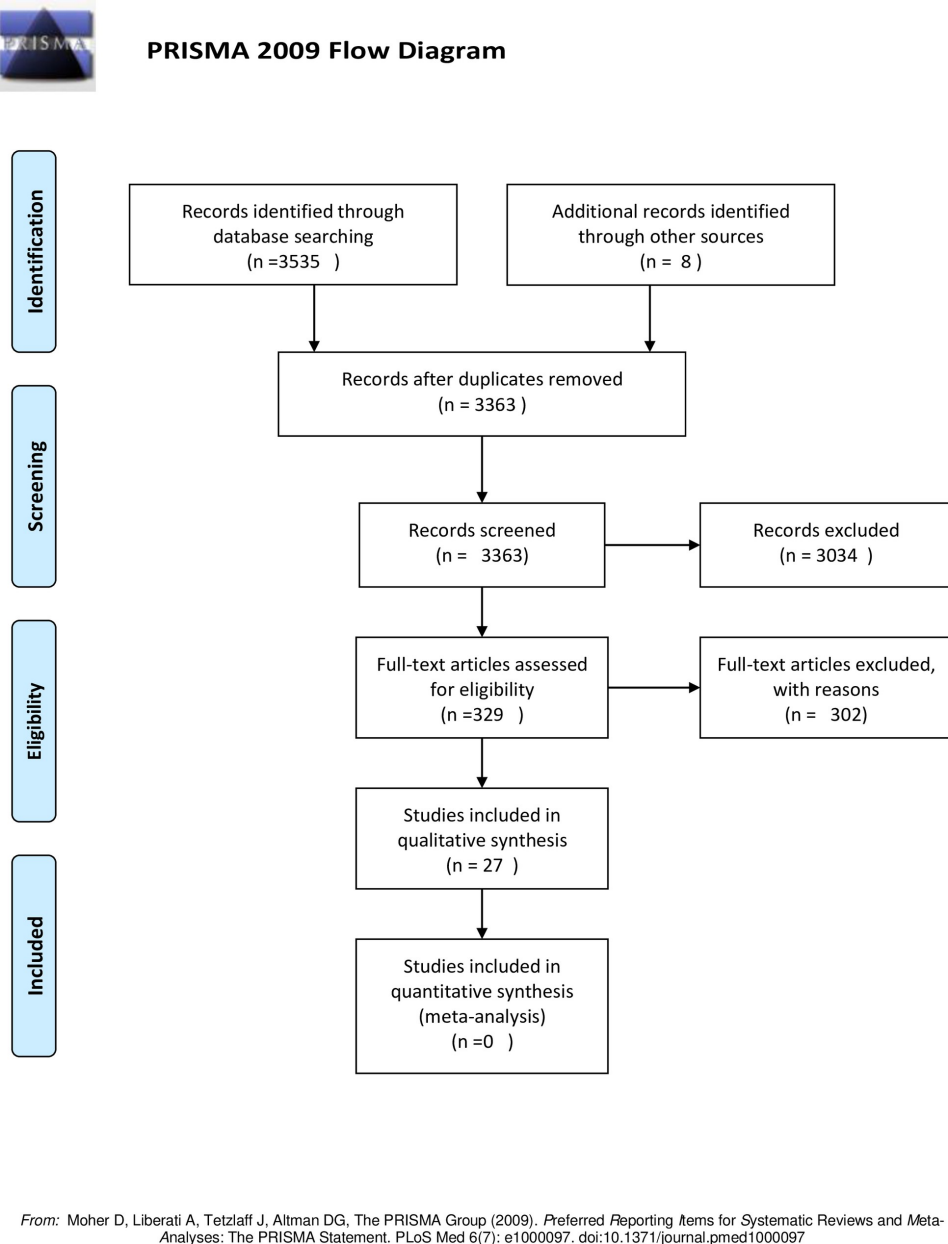
Flow diagram of the literature search according to the PRISMA statement.

The electronic search of five databases resulted in 3,535 studies. Of these, 329 were found eligible for a full-text review and 18 met our inclusion criteria. With an additional 8 studies found by manual screening, a total of 26 studies were included in our review. The selection process of the included papers is presented in Fig 1.

### Study characteristics

All 26 of the included studies were quantitative (22 were cross-sectional, 3 were cohort, and 1 was case-control) and all were written in English. Of these studies, 19 studies were conducted in the United States and 7 were conducted in other countries, including Canada, Israel, Netherlands, Germany, Sweden, and China. The characteristics of the studies are presented in Table 2.

**Table 2:** Characteristics of included studies.

Author Year	Host Country & Participants	Age	Study Type and Sample	Social support Measure	Type of social support	Association with Oral Health Outcome	Results
Arcury et al.[30] 2013	USA –635 white, African American, American Indian	60 and older	Cross sectional–Random	Social engagement; Social network size	Structural	Self-rated oral health, number of teeth; Number of oral health problems	-Social engagement: (+) number of teeth; (-) oral health problems; (+) high self-rated oral health - Social network size: (0) self-rated oral health; (0) oral health problems
Brzoska et al. [31] 2017	Germany -41,220 non- immigrants and Immigrants	18 years or older	Cross sectional–Random	Oslo-3 Social Support Scale	Perceived	Preventive dental care utilization	- Immigrants’ Poor social support: (-) dental care utilization
Burr et al.[32] 2012	USA– 2,978 African Americans and Hispanics	65 and older	Longitudinal–Random	Social integration indicators: Marital status; Self-reported frequency of interactions with children, relatives, friends; Self-reported feelings of loneliness; Social participation in religious services or volunteer work; Neighborhood social cohesion Social support indicators: Perceived social support; Actual social support (financial support); Child geographic proximity	Perceived + Actual + Structural + Functional	Dental care utilization	- Social integration: (+) visited a dentist in the past 2 years - Perceived social support: (0) Dental care utilization
Calvasina et al.[33] 2015	Canada - 3976 European origins, Arabic, African, Middle Eastern, South Asian,Chinese, East Asian, Latin American, and Caribbean	15 and older years	Longitudinal—Random	Three variables were used to assess social support: Having relatives in Canada; Social group membership; Frequency of visiting relatives in Canada	Structural	Self-related oral health	-Social support: (+) Self-reported dental problems
Documet et al.[2] 2018	USA– 140 Latino immigrants	27 years	Cross sectional—Convenience	Standardized four-item social support; Emotional; informational, Tangible; Affectionate support; Positive social interaction	Perceived + Functional	Dental care utilization in the past year	- Social support: (+) dental care utilization
Duijster et al.[34] 2014	Netherlands- 630 Children non- immigrants and Immigrants	6 years	Cross sectional—Random	GVL (translation ‘Family Questionnaire’) It assesses family functioning on five dimensions; responsiveness, communication, organization, partner-relation and social network.	Perceived	Dental caries; Oral hygiene behavior	-Poor Social support:(+) dental caries; (-) Oral hygiene behaviour
Duijster et al.[35] 2014	Netherlands- 630 children4 non- immigrants and Immigrants	5–6 years	Cross sectional- Random	GVL (translation ‘Family Questionnaire’) It assesses family functioning on five dimensions; responsiveness, communication, organization, partner-relation and social network.	Perceived	Dental caries; Oral hygiene behavior	-Family perceived social support: (+) children start brushing at a younger age; (-) dental caries
Finlayson et al.[36] 2007	USA- 719 African–American mother–child dyads	Mothers’ ages averaged 28 years; children’s age 1–5 years	Cross sectional- Random	Mother’s instrumental social support	Perceived	Dental caries	-Mother’s instrumental social support: (0) children’s caries
Gao et al.[37] 2014	China– 122 Indonesian domestic helpers	20–59 years	Cross sectional- Random	social connections	Structural	Dental caries	-Domestic helpers with family members or relatives in Hong Kong: (+) carious teeth
Graham et al.[38] 2005	USA - 810 immigrants from Cuba, Nicaragua, Colombia, and Puerto Rico	18 years or older	Cross sectional- Random	Modified version of the MacArthur Scale of Subjective Social Status	Perceived	dental care utilization	- Respondents perceived their social status in the United States to be higher than that in their community: (+) dental care utilization
Gironda et al.[39] 2013	USA- 2,598 Hispanic, white, African American, others	40 years and older	Cross sectional- Random	Five NHANES variables ^50^ to measure social support Marital status; Emotional support; Financial support; Number of close friends; Years lived in neighbourhood	Perceived + Structural	Dental care utilization	-Fewer number of close friends and low financial support: (-) dental care utilization and self-rated dental visits - Marital status, emotional support, or years lived in the neighborhood: (0) timely or self-care–related dental visit.
Jang et al.[40] 2014	USA– 209 Korean Americans	60 years or older	Cross sectional–Convenience	Three items from the Lubben’s Social Network Scale; Number of family or relatives seen at least once a month; Frequency of contact; The number of family or relatives the participants felt close to	Structural	Dental care utilization; Unmet dental needs; Self-rated oral health	- Family network: (+) dental care utilization; (-) Unmet dental needs; (+) self-rated oral health
Kallestal et al.[41] 2000	Sweden- 3370 Swedish, Nordic/ European, Eastern Europe, other	12 years	Intervention- Convenience	Close friends; Feeling of loneliness; Time spent with family and friends	Actual + structural	Oral health behaviour	-Poor social support: (+) poor oral health behaviour
Kamimura et al.[42] 2013	USA -187 (45 US born and 37 non-US born)	18 years or older	Cross sectional- Convenience	Medical Outcomes Study Social Support Survey (MOS-SSS) ^44^ for emotional social support	Perceived	Oral health related quality of life	- Social support among US born participants: (-) OHRQoL - Social support among non-US born participants: (+) OHRQoough
Kim et al. [43] 2005	USA- 320 Mexican, Puerto Rican, other Hispanic immigrants’ mothers	4–8 years	Cross sectional -Random	Acculturation index (Marin short acculturation scale, focuses on language proficiency, preference, and ethnicities of friends in the respondent’s social network.)	Actual	dental care utilization	- Social support: (+) dental care utilization
Maida et al. [44] 2013	USA- 5,014 African, Americans, Mexican Americans	20 years or older	Cross sectional—Random	Emotional support; Financial support	Actual + Functional	Oral Health Related Quality of Life (OHRQoL)	-Emotional support: (0) OHRQoL -Financial support: (+) OHRQoL.
Maupome [45] et al. 2016	USA -332 Latino immigrants	18–70 years	Cross sectional—Convenience	the Oral Health Matters (OHM)	Structural	Number of months since last dental visit; Main reason for last dental visit	-Educated oral health discussants in personal networks: (+) level of Education, which is associated with having more knowledge about dental services or oral hygiene practices. - Having any network member: (+) preventive-oriented dental care
Maupome et al.[46] 2016	USA - 332 Latino immigrants	18–70 years	Cross sectional—Convenience	the Oral Health Matters (OHM)	Structural	Frequency of discussion of oral health problems	- Having large network: (+) discussions about oral health problems - Having alters with more dental knowledge: (+) discussion of oral health issues in the preceding 12 months
Nahouraii et al. [18] 2008	USA– 174 mothers of Latino children	Younger than 11 years	Cross sectional–Convenience	The Spanish-language ethno-survey questionnaire to measure four dimensions of social support received by mothers.	Actual	Child dental care utilization	-Social support: (+) dental care utilization
Pullen et al.[47] 2017	USA- 332 Mexican-American immigrants	average is 40 years	Cross sectional—Convenience	Six network characteristics were examined: Network size; Network closeness; Influence of network norms on dental behavior; Strength of the alters may exert on egos in terms of oral health help-seeking; Participants report the amount they talk with alters about dental issues; ^48^The extent to which networks “push” egos to respond to dental problems	Actual + Structural	Dental care utilization; Dental knowledge; Dental treatment	-Large network size and frequency of discussion with ties regarding acute dental problems: (+) dental care utilization in the past year; (+) dental knowledge; (+) having dental treatment -Network hassling regarding dental issues:(-) dental care utilization
Sabbah et al.[48] 2011	USA- 1632 White,African Americans, Mexican Americans, other Hispanic	60 years or older	Cross sectional—Random	Social networks: Number of close friends; Marital status Social support: Whether the participants needed more emotional help during the past year	Actual+ structural+ Functional	Periodontal disease; Dental care utilization	-Social Network: (-) prevalence of periodontitis; (+) dental care utilization; (-) loss of periodontal attachment -Social support: (+) dental care utilization; (0) periodontitis or the extent of loss of periodontal attachment, (0) moderate periodontitis
Stapleton et al.[49] 2015	USA-1444 Black/African American men	18 years or older	Cross sectional - Convenience	Self-reported frequency of social/emotional support	Actual + Functional	Dental care utilization	-Having always/usually social support: (+) dental care utilization during the past year
Tellez et al.[50] 2006	USA-1005 African American caregivers of children under age six	14–70 years	Cross sectional -Random	Having instrumental or emotional support	Actual + Functional	Dental caries	-Caregivers who have more religious involvement and reported having instrumental and emotional social support: (-) untreated decayed surfaces
Vered et al.[51] 2011	Israel-340 Ethiopian immigrants	18–75 years	Longitudinal -Convenience	Social support scale for immigrants from Israel ^29^ (instrumental and emotional social support)	Perceived	Dental caries; Periodontal health	- Social support: (-) Dental caries; (0) periodontal disease
Wu et al.[52] 2011	USA- 4,859 Black, Hispanic, and White adults	60 years or older	Cross sectional—Random	Marital status; Number of close friends or relatives; Self-perception of whether someone else would provide financial support, if needed	Perceived + structural	Self-rated oral health; Dental care utilization; Dental caries	-Having a high number of friends: (+) self-rated oral health
Wu et al.[24] 2005	USA- 477 Chinese and Russian-speaking immigrant elders	60 years or older	Cross sectional—Convenience	frequency of seeing friends and family members	Structural	Dental care utilization;	-Social Support: (+) dental care utilization and dental treatment among Chinese speaking elders; (0) dental care utilization and dental treatment among Russian speaking elders
Wu et al.[53] 2011	USA- 4,355 non-Hispanic whites, Hispanic blacks, and Mexican-Americans	60 years or older	Cross sectional- Random	Marital status; Number of close friends or relatives; Self-perception of whether someone else would provide financial support, if needed.	Perceived + structural	Dental caries; Dental care utilization; Dentulous vs. Edentulous	-Social support of dentate individuals: (-) number of missing and filled teeth; (0) number of decayed teeth -Social support of Edentulism: (0) number of missing, decayed, and filled teeth.

(+) positive correlation; (-) negative correlation; (0) no correlation

The sample size of the included studies ranged from 122 [37] to 41,220 participants [31]. Social support level was assessed by using various social support indicators such as social network size [30, 47, 48, 52–54], social engagment, connection, and integration [24, 30, 32, 37, 47, 54], emotional support [2, 44, 48, 49], financial support [44, 52, 53] and instrumental support [36], or by adapting certain scales [18, 31, 34, 35, 40–43, 45, 51, 55].

### Statistical analysis used

Among the included studies, bivariate analysis was used in 25 of them. The applied tests included Rao-Scott chi-square tests [30], weighted frequencies/percentages[30], Pearson’s chi-square tests [18, 31, 37, 38, 41, 49], Wilcoxon-Mann-Whitney tests [31], Fisher’s exact chi-square test [51], the Sidak test [51], the Kruskal-Wallis test [35], the Mantel-Haenzel v2-test [35], analysis of variance (ANOVA) [42, 50, 53], and the Spearman correlation [52]. One study used univariate logistic regression instead of the bivariate test [2].

Next, multivariate analysis was applied among the included studies to determine the association between social support, oral health status/behavior, and controlling factors. Twenty studies used multivariate logistic regression [2, 18, 24, 31, 35–44, 46, 48, 49, 51, 53, 54], while other studies used multiple linear regression [30, 51]], binominal logistic regression [32], negative binominal regression [46], hierarchical linear models [50], and hierarchical block design in multivariate analyses [52]. A generalized estimating equation approach was used by one study to estimate the probability of reporting dental problems among immigrants, taking into consideration individual heterogeneity and controlling for the individual stock of independent variables [56]. In addition, one study applied structural equation modelling, which is a statistical technique that integrates factor analysis and multiple regression analysis and permits the simultaneous testing of inter-relationships among a number of potentially interdependent variables [34].

### Social support and oral health

#### Dental care utilization

The association between social support and dental care utilization was the most addressed oral health related factor among the included studies. Fifteen studies (58%) determined the effect of social support on dental care utilization [2, 18, 24, 31, 32, 39, 43, 46, 48, 49, 52–55, 57]. Immigrants and ethnic minorities who were socially integrated into a large network and received social support from individuals around them had visited the dental office in the past 1–2 years for treatment and preventive purposes [2, 24, 31, 32, 39, 43, 45, 48, 49, 54, 55, 57]. In addition, mothers who were part of a social network and had received social support reported taking their children to the dentist for treatment and preventive appointments more frequently (starting from a young age) compared with children whose mothers had not received any form of social support [18, 43]. In particular, functional support that was provided to mothers by family members or friends in the form of instrumental, influential, emotional and material aid was strongly associated with dental care use [18]. In contrast, children’s dental care utilization was not affected by social support (specifically, instrumental support) that was offered by dental clinics or hospitals [18]. While financial support was positively associated with dental visits, emotional support was not in one study [39]. Another study, immigrants who received financial support were less likely to have visited a dentist [32] and 2 studies reported no significant correlation between dental care utilization and level of social support in two of the studies [52, 53].

#### Oral health problems

Oral health problems, including dental caries and periodontal disease, were addressed by eleven studies [30, 34–37, 48, 50–53, 56]. One study assessed oral health problems from a broader perspective by investigating the effect of social support on participants’ self-reports of oral pain, denture problems, sore or bleeding gums, and dry mouth [30]. The findings showed that social integration was positively associated with fewer of these problems, while social network size did not attain statistical significance [30]. However, in another study, social support was reported to be associated with a high rate of dental problems [56].

The association between periodontal health and social support was reported by two studies [48, 51]. Although having a large social network was inversely associated with periodontal attachment loss [48], social support (including emotional support) was not significantly related to the extent of periodontal disease [48, 51].

Nine studies examined the relationship between social support and dental caries [34–37, 50–53, 57]. The effect of a high level of social support was associated with a reduced number of teeth with dental caries; this included adults as well as children, the latter who received social support indirectly through their parents’ social support [34, 35, 50, 51, 57]. Emotional and instrumental support were significantly associated with a lower number of carious teeth [50]. Surprisingly, domestic Indonesian helpers who were living in Hong Kong with their family members had increased prevalence of dental caries [37] and social support was not significantly related to the number of carious teeth in another two studies [52, 53].

#### Self-rated oral health and oral health-related quality of life

The association between social support and self-rated oral health was assessed by four studies [30, 40, 52, 56]. Social integration and having a high number of close friends or family members had a significant impact on immigrants’ and ethnic minorities’ self-rated oral health [30, 40, 52, 56]. Furthermore, two studies examined the relationship between oral health-related quality of life (OHRQoL) and social support (specifically, emotional and financial support) [42, 44]. Interestingly, neither study showed any significant association between emotional support and OHRQoL [42, 44], whereas financial support was positively associated with OHRQoL scores [44].

#### Oral health knowledge and behaviors

Two studies reported that immigrants and ethnic minorities who had a large network and more frequent discussions were more knowledgeable about dental care services and oral health knowledge, which consequently led to increased dental care utilization [45, 54]. In addition, three studies investigated the association between social support and oral health behaviors [34, 35, 41]. They found that children of families who had a strong social network that they could rely on (such as relatives or friends) had more favorable oral hygiene behaviors such as brushing at an early age [35]. In contrast, poor social support was indirectly related to poor oral health behaviors due to parents’ low level of education or individuals with low self-esteem [34, 41].

### Risk of bias in the included studies

Overall, the studies included in this systematic review attained medium to high methodological quality according to the grading method used [25, 54]. Table 3 presents the quality assessment of the included papers.

**Table 3:** Critical appraisal for quantitative studies.

Author Year	Selection (Max 5 stars)				Comparability (Max 1 stars)	Outcome (Max 3 stars)		Total score (max of 10)
	1. Representativeness of the sample	2. Sample size	3. Non–respondents	4. Social Support Tool	1.Participants in outcome groups are comparable	1. Assessment of the outcome	2.Statistical test	
	a) Truly representative of the average in the target population. * (all participants or random sampling) b) Somewhat representative of the average in the target population. * (non-random sampling) c) No description of the sampling strategy.	a) Justified and satisfactory. * b) Not justified.	a) Comparability between respondents and non-respondents characteristics is established, and the response rate is satisfactory. * b) The response rate is unsatisfactory, or the comparability between respondents and non-respondents is unsatisfactory. c) No description of the response rate or the characteristics of the responders and the non-responders.	a) Validated measurement tool. ** b) Non-validated measurement tool, but the tool is available or described.* c) No description of the measurement tool.	a) The study controls for the most important factor (select one). * b) The study control[s] for any additional factor. **	a) Independent masked. ** b) Self report. * c) No description.	a) Clearly described and appropriate, and the measurement of the association is presented, including confidence intervals and the probability level (p value). * b) The statistical test is not appropriate, not described or incomplete.	
Arcury et al.[30] 2013	a*	*	c	*	**	*	*	7
Brzoska et al. [31] 2017	a*	b	c	*	**	*	*	6
Burr et al.[32] 2012	a*	*	c	*	**	*	b	6
Calvasina et al.[33] 2015	a*	*	c	*	**	*	*	7
Documet et al.[2] 2018	b*	*	c	*	**	*	*	7
Duijster et al.[34] 2014	a*	*	c	*	**	*	*	7
Duijster et al.[35] 2014	a*	*	c	*	**	*	*	7
Finlayson et al.[36] 2007	a*	*	c	*	**	**	*	8
Gao et al.[37] 2014	a*	b	c	*	**	**	*	7
Graham et al.[38]2005	a*	*	c	*	**	*	*	7
Gironda et al.[39] 2013	a*	*	c	**	**	*	*	8
Jang et al.[40] 2014	b*	b	c	*	**	*	*	6
Kallestal et al.[41] 2000	b*	*	c	*	**	*	*	7
Kamimura et al.[42] 2013	b*	b	c	**	**	*	*	7
Kim et al. [43] 2005	a*	*	c	*	**	*	*	7
Maida et al. [44] 2013	a*	b	c	*	**	*	*	6
Maupome [45] et al. 2016	b*	b	c	*	**	*	*	6
Maupome et al.[46] 2016	b*	b	c	*	**	*	*	6
Nahouraii et al. [18] 2008	b*	b	c	*	**	*	*	6
Pullen et al.[47] 2017	b*	b	c	*	**	*	*	6
Sabbah et al.[48] 2011	a*	b	c	*	**	**	*	7
Stapleton et al.[49] 2015	b*	b	c	*	**	**	*	7
Tellez et al.[50] 2006	a*	*	c	*	**	**	*	8
Vered et al.[51] 2011	b*	*	c	**	**	**	*	9
Wu et al.[52] 2011	a*	*	c	*	**	*	*	7
Wu et al.[24] 2005	b*	b	c	*	**	*	*	6
Wu et al.[53] 2011	a*	*	c	*	**	**	*	8

A study can be awarded one star

“*” or maximum of two stars

“**” (representing “yes”) for each numbered item within the selection, comparability, and outcome categories.

## Discussion

Social life changes after migration play a crucial role in immigrants’ and ethnic minorities’ health, including their oral health. Therefore, this paper has systematically reviewed the literature on the associations between social support and oral health outcomes among this population, finding positive associations. Immigrants and ethnic minorities who experience social support either from their close surroundings (e.g., family members and friends) or from greater society had increased dental care utilization and improved oral health status, including caries level and periodontal disease. In addition, social support had a positive influence on self-rated oral health and oral health-related quality of life. Immigrants and ethnic minorities with strong social ties were also more knowledgeable about oral health and oral health care.

Increased dental care utilization among different ethnic groups was the most noticeable dental-related change as a result of social support [30]. In addition, social support promoted medical care utilization, physician contact, mental health services, and formal home care services among the elderly from different ethnicities [58–61]. Having a large network was also associated with ethnic minorities’ pediatric medical care utilization [62]. A possible explanation is that the process of care-seeking may be influenced by different types of functional support [32]. For instance, through informational and instrumental supports, individuals will receive health information and help with booking doctors’ appointments and transportation [18].

Surprisingly, less frequent dental care utilization was seen among some immigrants who received financial support [32]. Perhaps individuals who are eligible for financial assistance are more financially distressed and therefore feel they need to spend the received money on housing and education rather than on dental or health problems [32]. Another explanation for reduced health care utilization might be that individuals who receive tangible support may lead to lower self-esteem and low responsibility with a high sense of dependency, which can result in less favorable health behaviors like visiting a dentist [32]. Interestingly, not only was increased social support positively associated with health care utilization, but lack of social support and feelings of loneliness also resulted in seeking health care for more interaction and socialization rather than the need for treatment among individuals from different ethnic groups [62–66].

The positive effect of social support was not only improvement in adults’ oral health but also improvement in children’s oral health indirectly through their parents, especially the mothers. Aspects such as increased dental care utilization, better adaptation to positive oral health behaviors, and decreased dental caries levels among children could be a reflection of increased opportunities and resources for mothers to receive help from their surrounding individuals and to access more information about oral health and oral health care services [67]. Furthermore, mothers who are socially integrated are more capable of making relationships with not only friends and relatives, but also professionals and community resources, which in turn can contribute to a more positive impact on their children’s oral health [67].

Although social support was positively related to oral health outcomes in the included studies, one study reported a higher caries rate among Indonesian helpers in Hong Kong who had been surrounded by family members [37]. As well, living for a longer time in a host country was associated with a high rate of dental problems [51, 56]. These problems may occur due to a variety of factors, including financial problems [68]. Additionally, it is possible that immigrants’ stressful changes that result from resettlement after migration may promote negative oral health behaviors, which consequently lead to increased dental problems through consumption of a sugary diet [69]. Facing psychological stresses (e.g., discrimination) in the host country could also be associated with poor oral health [51, 56]. Another explanation is that immigrants who come from a country where poverty is common may simply be more susceptible to poor oral health [37].

Four studies explicitly compared between immigrant and non-immigrant populations on the impact of social support on oral health [31, 34, 35, 42]. Immigrants had lower level of social support compared to their counterparts and were 36% lower than non-immigrants in utilizing dental care utilization [31]. Moreover, being an immigrant child was associated with increased possibility of being part of a family receiving less social support [35]. In contrast, oral health related quality of life was reported better among non-US born when compared to US born participants [42]. However, in another study, no clear comparison was reported between immigrants and non immigrants [34]. These contradictory findings may be explained partially by the differences in predisposing factors, including sex, age, socioeconomic status, and marital status, and in enabling factors, such as type oh dental coverage, social support, place of resident, and type of residents [31, 70–73]. Furthermore, the level of social support act as a mediator to oral health outcomes [35, 42].

These associations between social support and oral health-related changes may be due to variations among ethnic groups in their adaptation toward social life changes after migration. For example, African Americans tended to have better social integration than Indian Americans or Whites [30], while older Chinese immigrants had more social interaction and were more frequently visited by their friends than did older Russian immigrants [24]. These variances may be due to some cultural background differences, the size of certain communities, or limited opportunity to participate in larger social events. Social support also varies among individuals based on their interpersonal characteristics, such as age, sex, socioeconomic status, cultural differences and type or stage of a disease, all of which may have an impact on health comes [74].

In measuring social support among the included studies, it was clear that there was a lack of attention paid to the multi-dimensional concept of social support [75]. Of the reviewed studies, 58% used unidimensional measures, such as number of close family members and friends, network closeness, marital status, social integration, frequency of visiting friends and relatives, perceived help and trust, and financial and emotional support. Other included studies (42%) adopted certain scales to measure social support [18, 31, 34, 35, 40–43, 45, 51, 55], some of which were validated tools, such as the Social Support scale for immigrants from Israel [51], the Medical Outcomes Study Social Support Survey (MOS-SSS) [42], and five NHANES variables [39].

Some of these scales were modified from original ones.[38, 40, 51] As an example, only three items from the original Lubben’s Social Network Scale was used, which has 6- or 12- item scales, and demonstrated an internal consistency (α = .75) among the included sample, which was satisfactory. [40] Furthermore, the Gezinsvragenlijst (GVL, translation; Family Questionnaire) that was used by several psychometric studies among large representative sample of the Dutch population to measure perceived social support was reported to be internally consistent (α = 0.83 to 0.95) and reliable over a period of 3–4 week. [35, 76] Six of the adopted scales assessed the perceived social support and four measured actual or received social support. Structural support was assessed by two of these scales and functional support by one. In addition to using unidimensional measures, some researchers tended to use general instruments to measure the construct without any specifications regarding a certain population or measuring a certain dimension of social support [75, 77, 78]. However, overreliance on unidimensional measures that make no distinction between different components of social support, structural and functional will lead to an incomplete assessment of social support [17, 75, 77–79].

The quality of the reviewed studies ranged from medium to high according to their total score based on the number of stars each study gained for sample and measurement tool selection, group compatibility, and outcome assessment. Study scores less than 3 were considered low quality, between 3 and 8 were medium quality and those above 8 were high quality. For example, a study conducted among the children of Latino immigrants to assess the association between dental care utilization and social support was considered medium quality due to issues such as non-random sampling, unjustified sample size, or lack of description of the response rate or responders’/non-responders’ characteristics [18]. In contrast, another study investigated the influence of psychological distress and social support on Ethiopian immigrants’ oral health. This study attained a high quality level, since the sample size was justified and satisfactory, a validated measurement social support tool was utilized, the study controlled for different factors, the oral health outcomes were assessed independently through dental examination, the statistical test was clearly described and appropriate, and the measurement of the association was presented, including confidence intervals and the probability level (p-value) [51]. The majority of the included studies were of medium quality due to the application of a non-validated social support measurement tool and self-reported oral health status/behaviors.

This review had some limitations that should be noted. We included studies that were conducted among immigrants and ethnic groups based on their reported definitions and main categories because it was difficult to differentiate between these two categories with hindsight [80]. In addition, the impact of social support on oral health may vary among these 2 groups due to the diversity at the individual and social level. Reasons for migration, origin and host countries, timing of migration within political, social environment, and individual life stage are examples of the. Therefore, it would be difficult, if not impossible, to include all these variables for analysis in one systematic review. Although there are some limitations in the quality appraisal tool used in this review, no validated methodological assessment tool has been designed specifically for observational studies.

### Conclusion

In this systematic review, the most addressed oral health outcomes among immigrants and ethnic minorities were dental care utilization (15 studies) and dental caries (9 studies). Social support was positively associated with dental care utilization in 13 studies and with dental caries in all 9 studies. Although, a lower number of studies investigated the associations between social support and periodontal disease, self-rated oral health, oral health-related quality of life, oral health knowledge, and oral health behaviors, the majority of them reported a relatively positive correlation. Further studies are needed, especially longitudinal and qualitative studies, to explore the detailed effect of social support on oral health by using validated multidimensional scales that are designed to address this association among certain population with the distinction between the functional and structural components to generate more comprehensive and comparable findings.

## Supporting information

S1 Table PRISMA 2009 checklist. (DOCX)
